# Sound Absorption of the Absorber Composed of a Shunt Loudspeaker and Porous Materials in Tandem

**DOI:** 10.3390/polym15143051

**Published:** 2023-07-15

**Authors:** Xin Li, Zhigang Cao, Lijun Xu, Bilong Liu

**Affiliations:** 1 Xinjiang Institute of Engineering, Urumqi 830023, China; 2 School of Mechanical & Automobile Engineering, Qingdao University of Technology, No. 777 Jialingjiang Road, Qingdao 266520, China

**Keywords:** shunt loudspeaker, porous materials, in tandem, common action, sound absorption

## Abstract

To investigate the sound absorption of the absorber composed of a shunt loudspeaker (SL) and porous materials (PM) in tandem, the normal absorption coefficients for six samples of different groups of parameters are measured using impedance tubes. It is shown that a composite structure consisting of a porous material, an air layer, a shunt loudspeaker, and an air layer arranged in sequence (PM + Air1 + SL + Air2) has the potential to achieve broadband sound absorption close to three octaves in the frequency range of 200–1600 Hz. To further explore the sound absorption mechanism of “PM + Air1 + SL + Air2”, a theoretical model based on the transfer matrix method is established, and a numerical model is built in the pressure acoustic module using COMSOL Multi-physics field software. The sound absorption coefficients and acoustic impedances predicted are in good agreement with those measured. The concerned “PM + Air1 + SL + Air2” with suitable parameters has two distinguishable sound absorption peaks in the low frequency domain and a well sound absorption spectrum similar to that of the porous material layer in the high-frequency domain. The reason for the superior sound absorption performance of “PM + Air1 + SL + Air2” lies in the fact that under the common action of the diaphragm’s mechanical vibration, the circuit’s damping loss, and the porous material’s viscous dissipation, the sound energy consumption is mainly dominated by SL in the low frequency domain and captured by PM in the high-frequency domain.

## 1. Introduction

Sound absorption is a common noise control method for restricting the propagation of sound waves along its pathway. Whether equipped with active devices, the sound absorption structure usually includes two types of passive and active absorbers [1,2]. Common passive sound absorbers mainly include the porous materials absorber and the resonant absorber, thin porous materials are suitable for mid–high-frequency sound absorption, and the classical micro-perforated plate (MPP), due to its Helmholtz resonance properties [3,4,5], is used for low frequency sound absorption still requires a large air cavity. Active sound absorbers are limited in applications due to their complex composition, insufficient stability, and high cost. With the development of shunt technology, research on the semi-active sound absorber based on the shunt loudspeaker has attracted much attention. The shunt loudspeaker is also known as an electroacoustic absorber, and its sound absorption process is as follows: when the moving coil loudspeaker is excited by the acoustic signal, which drives the diaphragm to vibrate, while the coil cuts through a magnetic field to generate electromotive force, so the acoustic energy is attenuated by the damping loss in the shunt circuit [6,7,8].

Previously, Lissek et al. [6] numerically and experimentally investigated the sound absorption of the shunt loudspeaker in open and short circuits. The results implied that the shunt loudspeaker could be used as a new acoustic liner device for noise reduction in aircraft cabins. Boulandet et al. [7], employing a response surface methodology, studied the optimization process of the shunt loudspeaker and established a multi-variate linear model by a series of designed experiments to analyze the effect of the relevant intrinsic parameters on the sound absorption performance of the shunt loudspeaker. Lissek et al. [9] elucidated the equivalence between shunts and active control by introducing a one-degree-of-freedom acoustic resonator accounting for both electric shunts and acoustic feedbacks. To quantify the sound absorption performance of the shunt loudspeaker, based on the equivalent electrical circuit theory, Cernik et al. [10] built the acoustic impedance model for a loudspeaker shunted by a negative impedance converter. Zhang et al. [11,12,13] studied in depth the acoustic absorption characteristics of a tunable acoustic impedance Shunted Electromagnetic Diaphragm (SEMD), and the analysis showed that under the electromechanical coupling effect, the system can achieve a vanishing acoustic reactance in a broad frequency band by connecting a well-designed shunt circuit. To investigate the optimal absorption of a shunt loudspeaker in a specific frequency range, Li et al. [14] performed a fully exhaustive backtracking algorithm to determine the optimal parameters for the loudspeaker and shunt circuit, and the results showed that a four-inch loudspeaker with a small force factor and low mechanical mass is able to have an average absorption coefficient above 0.65 at 100–450 Hz.

Based on the adjustable acoustic impedance of the shunt loudspeaker, a variety of composite structures with the shunt loudspeaker as the substrate for the expansion of the sound absorption bandwidth are proposed. Tao et al. [15,16] studied the sound absorption of a composite structure MPPSL of a micro-perforated plate backed by a close-box loudspeaker with a shunted circuit. The numerical and experimental results showed that compared to the classical MPP in the same space size, the designed MPPSL has an additional absorption peak from the shunt loudspeaker to extend the low frequency absorption range. Zhang et al. [17] designed a perforated membrane–loudspeaker composite structure, placing a loudspeaker at the rear end of the perforated membrane to enhance the low-frequency sound absorption of the single perforated membrane resonance structure. Rivet et al. [18] proposed three multi-degree-of-freedom electroacoustic systems through coupled resonators to achieve a multi-resonance enhanced sound absorption for low-frequency. Cong et al. [19,20,21] designed thin multi-tone sound absorbers through a loudspeaker connected with a multi-resonant shunt circuit or based on an array of shunted loudspeakers under the normal or random incidence for low frequency sound absorption. For the insufficient sound absorption for a small-scale loudspeaker, Li et al. [22] proposed a parallel structure SLPP of a shunted loudspeaker and a perforated plate with a common air cavity. An SLPP with a 2-inch shunt loudspeaker can attain three resonance peaks with quasi-perfect sound absorption coefficients, compared to a single shunt loudspeaker, its average sound absorption is increased more than 3 times in 100–250 Hz. In addition, Cao et al. [23] designed a hybrid absorber HSLPP consisting of a shunt loudspeaker and perforated plates arranged in series and in parallel; the results showed that the proposed HSLPP has an optimized average absorption coefficient of 0.87 in the 200–1000 Hz.

A lot of research has been done on the sound absorption performance of the composite structure the of shunt loudspeaker and perforated plate; however, there are few reports on the sound absorption of the composite structure made up of the shunt loudspeaker and porous material in tandem. In this paper, first, the sound absorption coefficients of six tandem samples with different parameters are measured and compared. Then, for “PM + Air1 + SL + Air2” with good capability of broadband sound absorption, the theoretical and numerical models are developed to predict its sound absorption performance and explore its sound energy dissipation mechanism. Moreover, the influence of the parameters of the shunt circuit and porous material on the sound absorption performance is also discussed.

## 2. Models and Methods

### 2.1. Experimental Measurement

**Figure 1:**
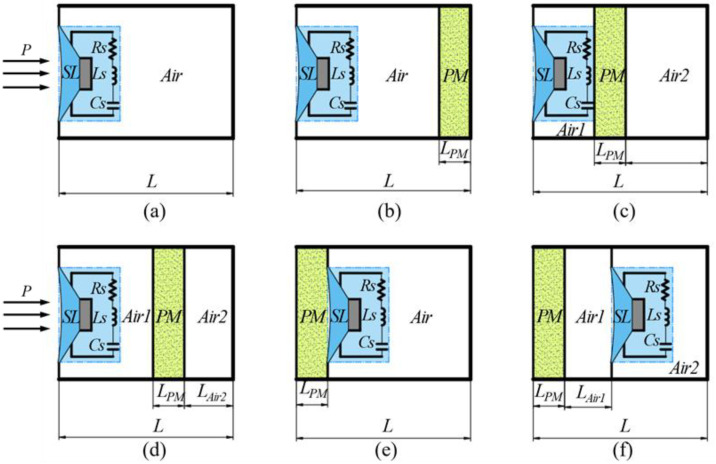
Schematic diagrams of six composite structures in tandem: (**a**) S1: SL + Air; (**b**) S2: SL + Air + PM; (**c**) S3: SL + PM + Air2 (PM is adjacent to the rear surface of SL); (**d**) S4: SL + Air1 + PM + Air2; (**e**) S5: PM + SL + Air (PM is adjacent to the front surface of SL); (**f**) S6: PM + Air1 + SL + Air2.

In the experiment, the sound absorption coefficients of six composite structures consisting of a shunt loudspeaker, porous materials, and air cavity in tandem were measured, and these schematic diagrams are shown in Figure 1. Figure 1a shows a simple and basic sound absorber made of a shunt loudspeaker backed by an air cavity, which is labeled as “SL + Air”. The serial structures containing a layer of porous material in the rear air cavity of the shunt loudspeaker under three arrangements are plotted in Figure 1b–d. As shown in Figure 1b, the structure is noted as “SL + Air + PM”, i.e., there is an air layer between the porous material layer and the shunt loudspeaker, and the porous material layer is tightly attached to the rigid wall. As shown in Figure 1c,d, two structures are classified as “SL + Air1 + PM + Air2”, among which, in Figure 1c, the porous material layer is attached directly behind the shunt loudspeaker; in Figure 1d, there is an air layer between the porous material layer and the shunt loudspeaker, as well as between the porous material layer and the rigid wall. The other two structures with the porous material layer located in front of the shunt loudspeaker are shown in Figure 1e,f. In Figure 1e, the porous material layer is placed closely in front of the shunt loudspeaker, ignoring the small air layer between them, and the structure is marked as “PM + SL + Air”; in Figure 1f, the porous material layer is placed in front of the shunt loudspeaker, and there is a measurable air layer between them, and the structure is marked as “PM + Air1 + SL + Air2”.The symbols *L*, *L_PM_*, and *L_Air_* indicate the overall longitudinal dimensions of the designed absorber, the thickness of the porous material layer, and the approximate thickness of the air cavity, respectively, and *Rs*, *Ls*, and *Cs* indicate the resistance, inductance, and capacitance of the shunt circuit connected to the loudspeaker.

**Figure 2:**
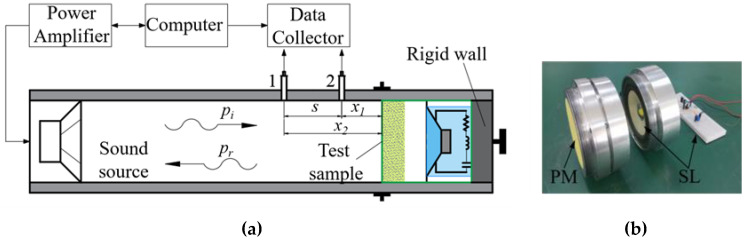
Diagram of the device for experimental measurements: (**a**) schematic diagram of impedance tube; (**b**) physical photo of the sample.

The schematic diagram of the impedance tube device used in the experiment measurement is shown in Figure 2a. The inner diameter of the SW422 impedance tube is 100 mm, and the measured sound absorption frequency is in the range of 63–1600 Hz. The left loudspeaker in the impedance tube emits white noise with uniformly distributed power spectral density, which is processed by the power amplifier PA50 to build a plane wave sound field in the impedance tube. The measured sample is installed in the right specimen tube, which is supported by a rigid wall, and the physical photos of the porous material layer and the shunt loudspeaker used are shown in Figure 2b. The diaphragm used in this work is a commercially available 4-inch, ultra-thin iron-framed and round loudspeaker suitable for the entire frequency range. The diaphragm’s diameter is approximately 87 mm, and the loudspeaker’s Thiele–Small parameters are obtained using a specialized Klippel electroacoustic tester. In addition, raw material tape or adhesive tape is usually used to ensure that there is no leakage of sound waves between the surrounding surface of the specimen and the inner wall of the impedance tube.

According to the two-microphone transfer function method in ISO 10534-2 [24], the basic process of sound absorption measurement in an impedance tube is described as follows: first, the sound pressure at position 1 and position 2 is collected using two ¼-inch microphones MPA416; the complex transfer function of the sound pressure at the two positions is calculated; then, the normal incident complex reflection factor is determined; and finally, the normal incident absorption coefficient and the surface acoustic impedance of the tested sample are obtained. The sound pressure at position 1 and position 2 is defined as:

(1) $$\begin{array}{l} p_{1}=P_{I}e^{jk_{0}x_{1}}+P_{R}e^{-jk_{0}x_{1}}\\ p_{2}=P_{I}e^{jk_{0}x_{2}}+P_{R}e^{-jk_{0}x_{2}} \end{array}.$$

The transfer functions of the total sound field, reflected acoustic wave, and incident acoustic wave at position 1 and position 2 are defined as

(2) $$\begin{array}{l} H_{12}=\frac{p_{2}}{p_{1}}=\frac{e^{jk_{0}x_{2}}+re^{-jk_{0}x_{2}}}{e^{jk_{0}x_{1}}+re^{-jk_{0}x_{1}}}\\ H_{I}=\frac{p_{2I}}{p_{1I}}=e^{-jk_{0}(x_{1}-x_{2})}=e^{-jk_{0}s}\\ H_{R}=\frac{p_{2R}}{p_{1R}}=e^{jk_{0}(x_{1}-x_{2})}=e^{jk_{0}s} \end{array},$$

where *P_I_* and *P_R_* are the amplitudes of incident and reflected acoustic waves; *x*_1_ and *x*_2_ denote the distances from position 1 and position 2 of the two microphones to the front surface of the test sample; and *s* is the distance between position 1 and position 2. By Equation (2), the reflection coefficient is deduced as

(3) $$r=\frac{H_{12}-H_{I}}{H_{R}-H_{12}}e^{j2k_{0}x_{1}}.$$

Following the law of energy conservation, the normal sound absorption coefficient α is expressed as

(4) $$\alpha =1-{\left|r\right|}^{2}.$$

The shunt loudspeaker component is a closed loop, which is composed of a moving coil loudspeaker and a simple RLC shunt circuit. A 4-inch commercial loudspeaker is used in the experiment; its equivalent mechanical mass, force resistance, force compliance, and force factor are written as Mm, Rm, Cm, and BL; and the diaphragm’s effective area is marked as *Sd*. The loudspeaker coil’s resistance and inductance are denoted as *Rc* and *Lc*; and the shunt circuit’s resistance, inductance, and capacitance are labeled as *Rs*, *Ls*, and *Cs*, respectively. The five non-acoustic parameters of the JCA model for the porous material layer are static flow resistance, σ; porosity, ϕ; viscous characteristic length, Λ; thermal characteristic length, Λ′; and tortuosity, $\alpha_{\infty }$. The loudspeaker’s Thiele–Small parameters are obtained by a specific Klippel electroacoustic instrument, and the JCA parameters of the porous material layer are determined based on the measured data via the inversion method. The prepared samples are cylinders with a cross-sectional diameter of 100 mm and a longitudinal length of 100 mm. The porous materials used in the four group samples are the common melamine foam and polyurethane foam; their thicknesses, $L_{PM}$, are 20 mm, 30 mm, 30 mm, and 40 mm, respectively; and the relevant parameters are listed in Table 1.

**Figure 3:**
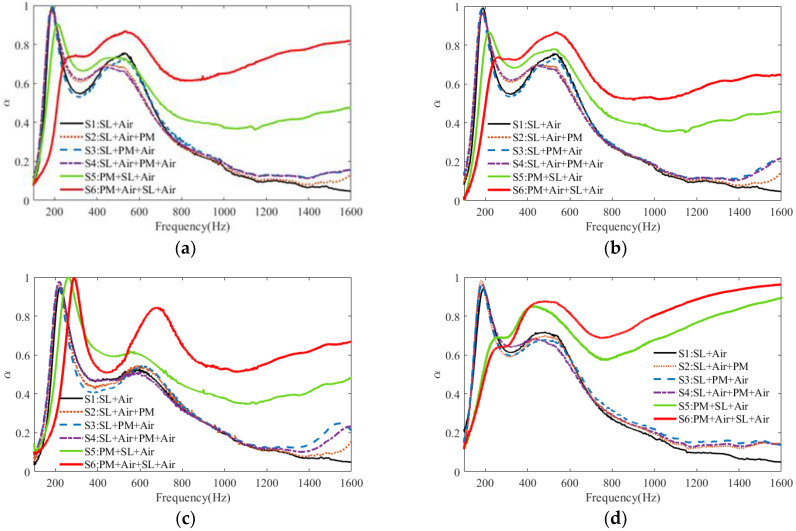
Plots of the measured sound absorption coefficients for six tandem structures under four groups of parameters: (**a**) G1; (**b**) G2; (**c**) G3; (**d**) G4.

The measured sound absorption coefficient curves of the six samples corresponding to the four groups of parameters are plotted in Figure 3a–d. At first, it can be seen that six structure samples of S1–S6 exhibit almost the same change in sound absorption for different structural parameters. Compared to S1, S2–S4 have almost no obvious sound absorption variation in the frequency range before the first peak, and there is only a slight increase in sound absorption at the first valley, the second peak, and in the higher frequency range of 1200–1400 Hz. In addition, compared with S1, for S5 and S6, the first resonance peak with a decreased sound absorption coefficient shifts to higher frequencies, but the absorption coefficient increases obviously in the higher frequency range of 600–1600 Hz, and the effective absorption frequency range is expanded to nearly three octaves. It can be found that when the porous material layer is located at the front of the shunt loudspeaker, the proposed tandem structure, especially S6, under the combined action of the electromechanical energy dissipation by the shunt loudspeaker at lower frequencies and the viscous energy dissipation by the porous material layer at higher frequencies, can exhibit an effective broadband sound absorption in the frequency range of 200–1600 Hz. Therefore, in the next sections, we focus on the sound absorption modeling and acoustic properties of ‘’S6: PM + Air + SL + Air”.

### 2.2. Model Building

In this section, to predict and clarify the sound absorption performance of S6, the theoretical and numerical models of acoustic impedance are established based on the transfer matrix method and COMSOL Multi-physics software, respectively.

**Figure 4:**
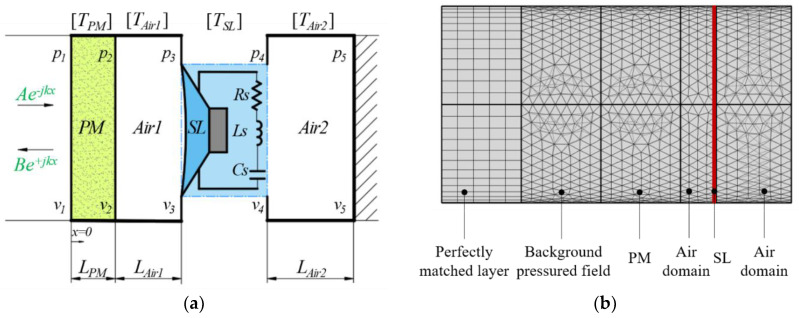
Model schematic of S6: PM + Air + SL + Air. (**a**) multiple-layer system; (**b**) numerical model.

Along the incident direction of sound waves, S6 is regarded as a tandem structure consisting of four units: porous material layer (PM), air cavity layer (Air1), shunt loudspeaker (SL), and air cavity layer (Air2) in a sequence. The transfer matrix method is widely used to estimate the sound absorption properties of the multi-layer acoustic structure because of its simplicity and high reliability. According to the transfer matrix method [25], for S6, its multiple-layer system is shown in Figure 4a, and the overall transfer matrix is expressed as

(5) $$\left[T\right]=\left[\begin{array}{cc} T_{11} & T_{12}\\ T_{21} & T_{22} \end{array}\right]=\left[T_{PM}\right]\left[T_{Air1}\right]\left[T_{SL}\right]\left[T_{Air2}\right],$$

where the overall transfer matrix [T] is a 2 × 2 square matrix; $T_{11}$, $T_{12}$, $T_{21}$, $T_{22}$ are the four pole parameters; $\left[T_{PM}\right]$, $\left[T_{Air1}\right]$, $\left[T_{SL}\right]$, $\left[T_{Air2}\right]$ are the unit transfer matrix of PM, Air1, SL, and Air2, which are written as

(6) $$\begin{array}{l} \left[T_{PM}\right]=\left[\begin{array}{cc} \cos(k_{PM}L_{PM}) & jz_{PM}\sin(k_{PM}L_{PM})\\ j\sin(k_{PM}L_{PM})/z_{PM} & \cos(k_{PM}L_{PM}) \end{array}\right]\\ \left[T_{Air1}\right]=\left[\begin{array}{cc} \cos(k_{air}L_{Air1}) & j\rho_{0}c_{0}\sin(k_{air}L_{Air1})\\ j\sin(k_{air}L_{Air1})/\rho_{0}c_{0} & \cos(k_{air}L_{Air1}) \end{array}\right]\\ \left[T_{SL}\right]=\left[\begin{array}{cc} 1 & Z_{SL}\\ 0 & 1 \end{array}\right]\\ \left[T_{Air2}\right]=\left[\begin{array}{cc} \cos(k_{air}L_{Air2}) & j\rho_{0}c_{0}\sin(k_{air}L_{Air2})\\ j\sin(k_{air}L_{Air2})/\rho_{0}c_{0} & \cos(k_{air}L_{Air2}) \end{array}\right] \end{array}.$$

$k_{PM}$, $z_{PM}$, and $L_{PM}$ are the complex wavenumber, the characteristic acoustic impedance, and the thickness of the porous material layer; $k_{air}$, $\rho_{0}c_{0}$, $L_{\mathrm{Air}}$ are the wavenumber, the characteristic acoustic impedance, and the equivalent thickness of the air cavity layer; and $Z_{SL}$ is the acoustic impedance of the shunt loudspeaker.

The melamine foam and polyurethane foam used are treated as porous materials with a rigid skeleton. According to the semi-empirical JCA model, the complex wave number and the characteristic acoustic impedance of the porous material layer are expressed as

(7) $$\begin{array}{l} z_{PM}=\sqrt{\rho_{PM}\cdot K_{PM}},\;k_{PM}=\sqrt{\omega \left(\rho_{PM}/K_{PM}\right)}\\ \rho_{PM}=\frac{\rho_{0}\alpha_{\infty }}{\phi }\left(1+\frac{\phi \sigma }{j\omega \rho_{0}\alpha_{\infty }}\sqrt{1+j\omega \rho_{0}\eta {\left(\frac{2\alpha_{\infty }}{\sigma \phi \Lambda }\right)}^{2}}\right)\\ K_{PM}=\frac{\gamma P_{0}}{\phi }{\left(\gamma -\left(\gamma -1\right){\left(1+\frac{8\eta }{j\omega \rho_{0}N_{\mathrm{Pr}}\Lambda \prime^{2}}\sqrt{1+j\omega \rho_{0}\eta \frac{N_{\mathrm{Pr}}\Lambda \prime^{2}}{16\eta }}\right)}^{-1}\right)}^{-1} \end{array}.$$

$\rho_{PM}$ and $K_{PM}$ are the equivalent dynamic density and the equivalent bulk modulus; ω is the angular frequency; $P_{0}$ is the ambient atmospheric pressure; η, γ, and $N_{\mathrm{Pr}}$ are the dynamic viscosity, specific heat ratio, and Prandtl number of the saturated air, respectively.

For the component SL, its acoustic impedance is made up of the diaphragm’s mechanical impedance, $Z_{m}$, and electrically-induced impedance, $Z_{e}$, which is written as

(8) $$\begin{array}{l} Z_{SL}=\left(Z_{m}+Z_{e}\right)\frac{S_{0}}{S_{d}}\\ Z_{m}=\frac{\left(R_{m}+j\omega M_{m}+{\left(j\omega C_{m}\right)}^{-1}\right)}{S_{d}}\\ Z_{e}=\frac{{\left(Bl\right)}^{2}}{\Delta R+j\omega \Delta L+{\left(j\omega \Delta C\right)}^{-1}}\frac{1}{S_{d}} \end{array}.$$

The total resistance, inductance, and capacitance in the shunt loudspeaker are defined as $\Delta R=R_{c}\pm R_{s}$, $\Delta L=L_{c}\pm L_{s}$, and $\Delta C=C_{s}$; $S_{d}$ and $S_{0}$ are the loudspeaker diaphragm’s effective area and the total cross-sectional area of the studied structure.

Then, the relationship of the sound pressure, $p_{1}$, particle velocity, $v_{1}$, on the left plane of PM and the sound pressure, $p_{5}$, particle velocity, $v_{5}$, on the right plane of the rigid wall is satisfied by

(9) $$\left[\begin{array}{c} p_{1}\\ v_{1} \end{array}\right]=\left[\begin{array}{cc} T_{11} & T_{12}\\ T_{21} & T_{22} \end{array}\right]\left[\begin{array}{c} p_{5}\\ v_{5} \end{array}\right].$$

In addition, at *x* = 0 on the left end plane, the sound pressure, $p_{1}$, and particle velocity, $v_{1}$, is expressed as

(10) $$\left[\begin{array}{c} p_{1}\\ v_{1} \end{array}\right]=\left[\begin{array}{c} A+B\\ \left(A-B\right)/\rho_{0}c_{0} \end{array}\right].$$

In addition, the right plane is a rigid wall; thus, $v_{5}=0$. Through Equations (9) and (10), the sound pressure reflection coefficient and the acoustic impedance of this structure are derived as

(11) $$\begin{array}{l} r=\frac{B}{A}=\frac{T_{11}/T_{21}-\rho_{0}c_{0}}{T_{11}/T_{21}+\rho_{0}c_{0}}\\ Z=\frac{T_{11}}{T_{21}} \end{array}.$$

Finally, the sound absorption coefficient of the structure S6 at normal incidence is written as

(12) α=1−r2=4Re(Z/ρ0c0)1+Re(Z/ρ0c0)2+Im(Z/ρ0c0)2=4R1+R2+X2,

where R=Re(Z/ρ0c0) and X=Im(Z/ρ0c0) are the specific acoustic resistance and acoustic reactance.

It has been confirmed that acoustic simulation using COMSOL Multi-physics finite element software can accurately and visually demonstrate the sound absorption mechanism [26,27,28,29]. First, we analyze the components of S6, which consists of a porous acoustic material layer, two air layers, and a shunt loudspeaker; the Pressure Acoustics, Frequency Domain Interface is chosen as the acoustic environment for the modeling of S6; and the profile of the three-dimensional model is shown in Figure 4b. The perfectly matched layer, background sound field, porous material layer, air domain, shunt loudspeaker, and air domain are set from left to right, where the porous material layer and shunt loudspeaker are defined using the “Porous-acoustics” node and “Interior Impedance” node, respectively. Then, the free tetrahedral meshes are constructed in the background sound field, porous material domain, and air domain, and the swept meshes are added in the perfectly matched layer, while the minimum mesh size is less than one-sixth of the minimum wavelength to ensure the accuracy of the calculation. Next, the solution is performed in the frequency domain under the study node. Finally, the post-processed and analyzed results from the acoustic simulation are performed in the results node.

**Figure 5:**
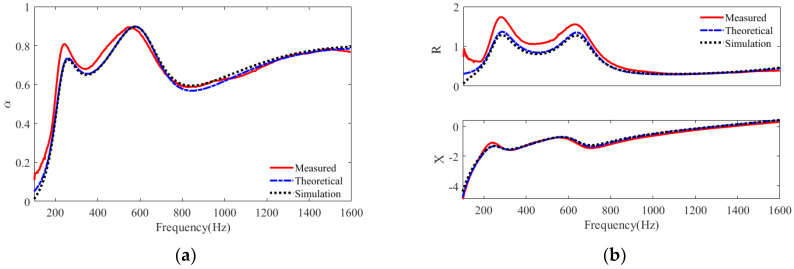
Plots of sound absorption for S6 by theoretical calculation and finite element simulation: (**a**) sound absorption coefficients; (**b**) specific acoustic impedances.

The relevant parameters of an S6 absorber containing a polyurethane material layer with a thickness of 40 mm are listed in Table 2. The absorption coefficients and acoustic impedances obtained from the measured, theoretical, and numerical models are plotted in Figure 5. First, it can be seen that the theoretical calculation results are in better agreement with the numerical calculation results. The error between the measured and predicted results is mainly around the first two peaks, which may be due to the deviation of the structural parameters and the installation conditions. As a whole, the developed theoretical model based on the transfer matrix and the numerical model in the pressure acoustic module can predict the sound absorption of the studied structure in a wide frequency range of 200–1600 Hz.

## 3. Results and Discussion

### 3.1. Analysis of the Sound Absorption Mechanism

**Figure 6:**
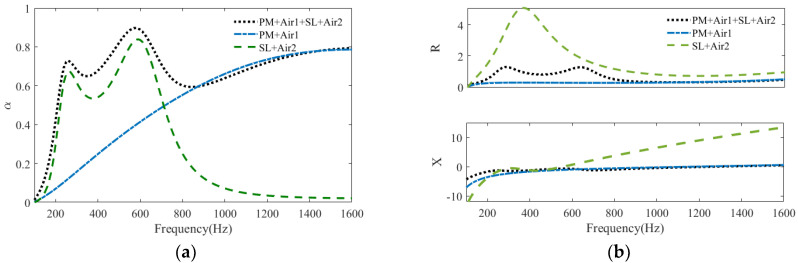
Sound absorption of “PM + Air1 + SL + Air2” and two components: “PM + Air1” and “SL + Air2”: (**a**) sound absorption coefficients; (**b**) normalized acoustic impedance.

Figure 6 plots the sound absorption coefficients and acoustic impedances of the tandem structure of “PM + Air1 + SL + Air2” and two components—“PM + Air1” and “SL + Air2” in the frequency range of 100–1600 Hz—and the related parameters are listed in Table 2. From the sound absorption coefficient curves, it can be found that at low frequencies, the first two peaks of “PM + Air1 + SL + Air2” are similar to those of “SL + Air2”, while at high frequencies, the absorption coefficient of “PM + Air1 + SL + Air2” almost coincides with that of “PM + Air1”. Moreover, unlike the under-resistance of “PM + Air1” and the over-resistance of “SL + Air2” in the frequency range of 200–800 Hz, “PM + Air1 + SL + Air2” has a normalized acoustic resistance that varies relatively gently around 1, and its normalized acoustic resistance is close to 1, which is similar to that of the “PM + Air1” in the frequency range of 800–1600 Hz. At the same time, “PM + Air1 + SL + Air2” has a normalized acoustic reactance approaching the zero line with a small slope change. Thus, compared with “PM + Air1”and “SL + Air2”, “PM + Air1 + SL + Air2” has a characteristic impedance that is more compatible with that of air in a wide frequency range; that is, for “PM + Air1 + SL + Air2”, its acoustic energy absorption at the lower frequencies is mainly dependent on the electromechanical resonance dissipation from the shunt loudspeaker, and its acoustic energy absorption at the higher frequencies is mostly attributed to the viscous dissipation from the porous material.

**Figure 7:**
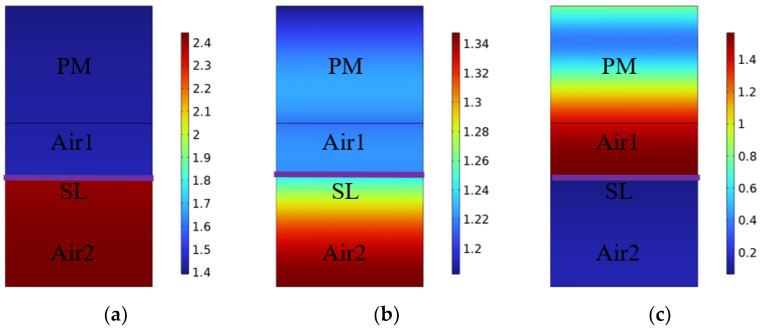
The distribution of normalized sound pressure at three frequencies of (**a**) 260 Hz, (**b**) 580 Hz, and (**c**) 1570 Hz.

Additionally, the distribution of normalized sound pressure at frequencies corresponding to three maximum absorption coefficients is shown in Figure 7a–c. At the first two peak frequencies of 260 Hz and 580 Hz, the normalized sound pressure increases from the top to the bottom; that is, low-frequency acoustic waves with a large wavelength can easily pass through the porous layer, and sound energy is consumed by the diaphragm vibration and the circuit damping; thus, the maximum normalized sound pressure appears at the cavity layer Air2 behind SL. At the higher frequency of 1500 Hz, the maximum normalized sound pressure is concentrated in the bottom position of PM and Air1; this is because the high-frequency acoustic waves are mainly trapped in the porous material. It is confirmed that “PM + Air1 + SL + Air2” can attain an effective broadband sound absorption under the combined effect of the porous materials and shunt loudspeaker.

### 3.2. Influence of the Parameters on Sound Absorption

**Figure 8:**
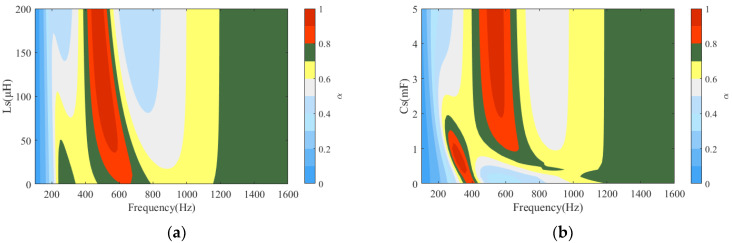

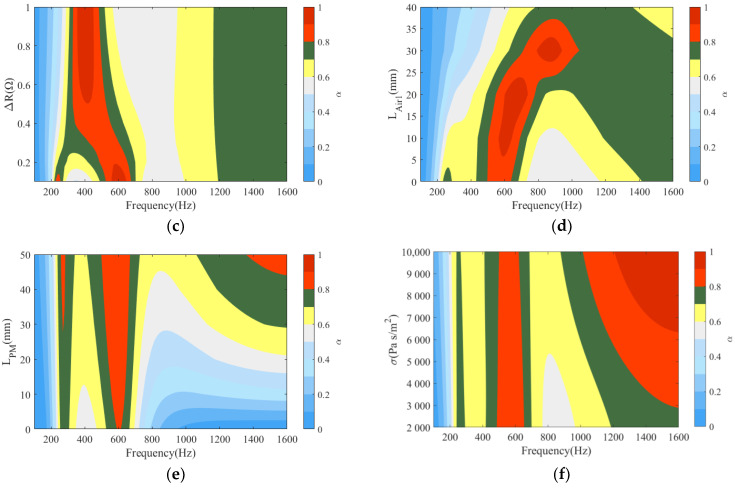
Variation of sound absorption for “PM + Air1 + SL + Air2” under different parameters: (**a**) inductance, Ls; (**b**) capacitance, Cs; (**c**) total resistance, ΔR; (**d**) thickness of Air1; (**e**) thickness of PM; (**f**) flow resistance of porous materials.

Figure 8a–d shows the absorption spectrum of “PM + Air1 + SL + Air2” with the variation of inductance, capacitance, total resistance in the shunt circuit, thickness of Air1 layer and PM layer, and flow resistance of the PM layer, respectively. From Figure 8a, as the additional inductance Ls increases from 0 μH to 200 μH, the sound absorption from SL is shifted toward lower frequencies, and the first peak and two valleys have significantly decreased absorption coefficients. From Figure 8b, as the additional capacitance Cs varies from 0 mF to 5 mF, the absorption peak generated by the shunt loudspeaker changes from one to two and then reduces to one, the sound absorption at the first peak changes clearly, while the sound absorption at the second peak changes relatively slowly, and there are two peaks with absorption coefficients greater than 0.6 at 0.8–2.6 mF. As the total resistance increases from 0 Ω to 1 Ω, two distinguishable absorption peaks in the low-frequency domain gradually converge into a single peak with an increased absorption coefficient and narrowed effective absorption bandwidth, and the sound absorption at the second valley moves to the lower frequency. From Figure 8a–c, it can be found that the first two peaks and two valleys on the absorption coefficient have obvious fluctuations with different inductance, capacitance and resistance. These three parameters in the shunt circuit have a significant effect on the sound absorption from the shunt loudspeaker in the low-frequency domain, while they have little effect on the sound absorption in the high-frequency domain from the porous material.

When the thickness of PM and the total longitudinal dimension of “PM + Air1 + SL + Air2” are fixed, from Figure 8d, as the thickness of Air1 increases from 0 mm to 40 mm, the sound absorption of the first peak becomes weaker, but the second peak and valleys have enhanced sound absorption. A proper Air1 layer not only makes the SL component attain two effective absorption peaks at low frequencies; it also promotes the PM component to have efficient sound absorption at high frequencies. When the thickness of Air1 and the total longitudinal dimension of “PM + Air1 + SL + Air2” remain unchanged, from Figure 8e, as the thickness of the PM increases from 0 mm to 40 mm, the overall sound absorption is significantly increased in 100–1600 Hz, i.e., adding a porous material layer in front of the SL component is helpful in achieving an enhanced sound absorption in a wide frequency range. Moreover, from Figure 8f, when the flow resistance is varied from 2000 Pa s/m^2^ to 10,000 Pa s/m^2^, there is a slight decrease in sound absorption at the two peaks, and the increased flow resistance can raise the absorption coefficient at the second valley, especially to improve the sound absorption capacity for PM. Therefore, for an appropriately high flow resistance, effective sound absorption is possible to extend to low and high frequencies.

## 4. Conclusions

In this paper, six tandem absorber samples containing a shunt loudspeaker, a porous materials layer, and an air layer were first prepared, and then their sound absorption coefficients were measured using an impedance tube based on the two-microphone transfer function method. Twenty-four test data show that at the same spatial scale, compared to the other five structures, “PM + Air1 + SL + Air2” is more likely to achieve an enhanced effective sound absorption close to three octaves at 200–1600 Hz. After that, to further describe the sound absorption characteristics of “PM + Air1 + SL + Air2”, the theoretical model using the sound transfer matrix method and the numerical model in the pressure acoustic module of the COMSOL Multi-physics field were developed, respectively. The potential sound absorption mechanism of “PM + Air1 + SL + Air2” is the fact that the acoustic energy absorption at the lower frequencies mainly depends on the electromechanical resonance dissipation by the shunt loudspeaker, and the acoustic energy absorption at the higher frequencies is mostly attributed to viscous dissipation by the porous material. Moreover, the parametric analysis illustrates that the sound absorption of the SL component is mainly related to the inductance, capacitance, and resistance in the shunt circuit, while the thickness of the porous material and the flow resistance of the porous material mainly affect the sound absorption of the PM component. The reasonably parameterized “PM + Air1 + SL + Air2” has two distinguishable peaks of high absorption coefficient in the low frequency domain and an absorption spectrum similar to that of the porous material layer in the high-frequency domain. In summary, “PM + Air1 + SL + Air2” has the potential to extend the low- or high-frequency absorption bandwidth for a single porous material layer or shunt loudspeaker on a small scale. This work provides a valuable means for the application of a small-scale loudspeaker and a thin porous material layer in low–mid-frequency broadband sound absorption.

## Figures and Tables

**Table 1:** Structural parameters of several groups of samples.

Group		Parameter					
G1	SL	$M_{m}$ (g)	$R_{m}$ (kg/s)	$C_{m}$ (mm/N)	BL (N/A)	$R_{c}$ (Ω)	$L_{c}$ (μH)
		2.711	0.448	0.626	1.339	3.77	72
		$R_{s}$ (Ω)	$L_{s}$ (μH)	$C_{s}$ (mF)	$S_{d}$ (m^2^)		
		−3.57	26.62	1.842	0.0059		
	PM (Melamine)	$\sigma (Pa\cdot s/m^{2})$	ϕ (%)	Λ (μm)	Λ′ (μm)	$\alpha_{\infty }$	*L_PM_* (mm)
		6883	99.6	179	318	1.05	20
G2	SL	$M_{m}$ (g)	$R_{m}$ (kg/s)	$C_{m}$ (mm/N)	BL (N/A)	$R_{c}$ (Ω)	$L_{c}$ (μH)
		2.711	0.448	0.626	1.339	3.77	72
		$R_{s}$ (Ω)	$L_{s}$ (μH)	$C_{s}$ (mF)	$S_{d}$ (m^2^)		
		−3.57	26.62	1.842	0.0059		
	PM (Polyurethane)	$\sigma (Pa\cdot s/m^{2})$	ϕ (%)	Λ (μm)	Λ′ (μm)	$\alpha_{\infty }$	*L_PM_* (mm)
		2368	98.45	185	206	1.06	30
G3	SL	$M_{m}$ (g)	$R_{m}$ (kg/s)	$C_{m}$ (mm/N)	BL (N/A)	$R_{c}$ (Ω)	$L_{c}$ (μH)
		2.711	0.448	0.626	1.339	3.77	72
		$R_{s}$ (Ω)	$L_{s}$ (μH)	$C_{s}$ (mF)	$S_{d}$ (m^2^)		
		−3.63	14.66	1.17	0.0059		
	PM (Polyurethane)	$\sigma (Pa\cdot s/m^{2})$	ϕ (%)	Λ (μm)	Λ′ (μm)	$\alpha_{\infty }$	*L_PM_* (mm)
		2368	98.45	185	206	1.06	30
G4	SL	$M_{m}$ (g)	$R_{m}$ (kg/s)	$C_{m}$ (mm/N)	BL (N/A)	$R_{c}$ (Ω)	$L_{c}$ (μH)
		2.711	0.448	0.626	1.339	3.77	72
		$R_{s}$ (Ω)	$L_{s}$ (μH)	$C_{s}$ (mF)	$S_{d}$ (m^2^)		
		−3.57	34.85	1.97	0.0059		
	PM (Melamine)	$\sigma (Pa\cdot s/m^{2})$	ϕ (%)	Λ (μm)	Λ′ (μm)	$\alpha_{\infty }$	*L_PM_* (mm)
		8102	99.36	102	146	1.02	40

**Table 2:** The related parameters of an S6 absorber with a polyurethane material layer.

*Mm* (g)	*Rm* (kg/s)	*Cm* (mm/N)	*BL* (N/A)	ΔR (Ω)	ΔL (mH)	*L_PM_* (mm)	*S_d_* (m^2^)
2.711	0.448	0.626	1.339	0.2	28.5	40	0.0059
ΔC (mF)	$\sigma (Pa\cdot s/m^{2})$	Λ (μm)	Λ′ (μm)	ϕ (%)	$\alpha_{\infty }$	L_Air1_ (mm)	L_Air2_ (mm)
1.795	2080	150	202	99.14	1.09	18	37

## Data Availability

All required data is in the text and there is no other data missed.
